# Improving quality of psychiatry training in Northern Ireland through the introduction of postgraduate education fellows

**DOI:** 10.1192/bjo.2021.366

**Published:** 2021-06-18

**Authors:** Catherine Boucher, Roisin Connolly, Michael Doris, Colin Gorman, Michael McMorran, Aisling Sheridan

**Affiliations:** 1 South Eastern Health & Social Care Trust; 2 Southern Health & Social Care Trust; 3 Belfast Health & Social Care Trust; 4 Northern Health & Social Care Trust; 5 Western Health & Social Care Trust

## Abstract

**Aims:**

To improve postgraduate psychiatry education and training in Northern Ireland.

**Background:**

Historically within Northern Ireland there has been a postgraduate Member of the Royal College of Psychiatrists (MRCPsych) teaching programme delivered to core trainees in preparation for MRCPsych examinations. There has been no official teaching programme for higher trainees. Northern Ireland Medical and Dental Training Agency (NIMDTA), in collaboration with the Royal College of Psychiatrists in Northern Ireland and all five Trusts developed the novel idea of introducing Postgraduate Education Fellows, to oversee and improve core training, and to develop a bespoke higher training programme.

The Postgraduate Education Fellows met to collate information from various sources in relation to issues within the current teaching programme and address these along with the development of new initiatives. The fellows further act as a point of contact for all trainees within their Trust to provide advice and support with education if needed.

**Method:**

One higher trainee was appointed to the role of Postgraduate Education Fellow in each Trust within the NIMDTA deanery for a term of 1 year.

The starting point was delivering the pre-established teaching timetable and gaining feedback from core trainees to identify areas for improvement. The next phase involved piloting traditional and contemporary methods of feedback. A further development was designing a mock paper A delivered under exam conditions. Two mock Clinical Assessment of Skills and Competencies (CASC) exams were organised under exam conditions, offering other trainees the opportunity to act as simulated patients and examiners.

The third aspect of this role involved creating a programme of higher trainee seminars. Baseline data were collated and identified key areas that higher trainees felt they needed further training and guidance in.

**Result:**

Using baseline data on the current teaching programme and from higher trainees as well as incorporating quality improvement methodology, we have been making small changes to each aspect of the teaching programme and evaluating the changes made. The feedback from trainees has been positive as evidenced by quantitative and qualitative feedback. 8 candidates sat our first mock CASC with a 100% pass rate in their MRCPsych CASC examination. There has been a positive response to the higher trainee seminar programme.

**Conclusion:**

This programme has produced good outcomes to date and sets foundations for the future development of post graduate psychiatry education in Northern Ireland.

